# Supplementary material for: SARS-CoV-2 mRNA vaccination elicits broad and potent Fc effector functions to VOCs in vulnerable populations
Source: medRxiv. 2022 Sep 18:2022.09.15.22280000. Preprint. [Version 1] doi: 10.1101/2022.09.15.22280000 (PMC9516864; doi:10.1101/2022.09.15.22280000)
Supplement: 1 [file NIHPP2022.09.15.22280000V1-supplement-1.pdf]

medRxiv preprint doi: https://doi.org/10.1101/2022.09.15.22280000; this version posted September 18, 2022. The copyright holder for this preprint (which was not certified by peer review) is the author/funder, who has granted medRxiv a license to display the preprint in perpetuity.

It is made available under a CC-BY 4.0 International license .

Supplemental Table 1. Fc detection and antigen reagents

Antigen	Source	Fc Detection	Source
SARS-CoV-2 S	Acro Biosystems SPN-C82E9	a- IgG	Southern Biotech 2048-09
SARS-CoV-2 S1	Acro Biosystems S1N-C52H3	a-IgG1	Southern Biotech 9054-09
SARS CoV-2 S2-P	Plasmid provided by Jason McLellan	a-IgG2	Southern Biotech 9070-09
SARS CoV-2 S-6P	Plasmid provided by Jason McLellan	a-IgG3	Southern Biotech 9210-09
SARS CoV-2 RBD	BEI Resources NR-52366	a-IgG4	Southern Biotech 9200-09
SARS CoV-2 S2	Immune Technology IT-002-034p	a-IgA	Southern Biotech 2050-09
SARS-CoV-2 S Alpha (B.1.1.7)	Sino Biological 40589-V08B6	a-IgM	Southern Biotech 9020-09
SARS-CoV-2 S Beta (B.1.351)	Sino Biological 40589-V08B7	FcγR2a	Boesch, et. al, 2014
SARS-CoV-2 S Gamma (P.1)	Sino Biological 40589-V08B8	FcγR2b	Boesch, et. al, 2014
SARS-CoV-2 S Delta (B.1.617.2)	Sino Biological 40589-V08B12	FcγR3a	Boesch, et. al, 2014
SARS-CoV-2 S Omicron (B.1.1.529)	Sino Biological 40589-V08H26	FcγR3b	Boesch, et. al, 2014
SARS-CoV-2 RBD Alpha (B.1.1.7)	Sino Biological 40592-V08H82		
SARS-CoV-2 RBD Beta (B.1.351)	Sino Biological 40592-V08H4		
SARS-CoV-2 RBD Gamma (P.1)	Sino Biological 40592-V08H86		
SARS-CoV-2 RBD Delta (B.1.617.2)	Sino Biological 40592-V49H-B		
SARS-CoV-2 RBD Omicron (B.1.1.529)	Sino Biological 40592-V08H121		
SARS-CoV S	Sino Biological 40634-V08B		
SARS-CoV S1	Sino Biological 40634-V08B		
MERS S	Sino Biological 40069-V08B-B		
MERS S1	Sino Biological 40069-V08B1		
OC43 S	Sino Biological 40607-V08B		
OC43 S-2P	Plasmid provided by Jason McLellan		

medRxiv preprint doi: https://doi.org/10.1101/2022.09.15.22280000; this version posted September 18, 2022. The copyright holder for this preprint (which was not certified by peer review) is the author/funder, who has granted medRxiv a license to display the preprint in perpetuity.

It is made available under a CC-BY 4.0 International license .

OC43 S2	Sino Biological 40069-V08B		
229E S	Sino Biological 40605-V08B		
229E S1	Sino Biological 40601-V08H		
HKU1 S	Sino Biological 40606-V08B		
HKU1 S1	Sino Biological 40602-V08H		
NL63 S	Sino Biological 40606-V08B		
NL63 S1	Sino Biological 40604-V08H		
